# Psychological Distress and coping strategies of patients with Chronic Diseases

**DOI:** 10.1192/j.eurpsy.2023.2104

**Published:** 2023-07-19

**Authors:** M. Theodoratou, C. Vassilopoulou, V. Giotsidi, G. Tsitsas, K. Flora, G. Kougioumtzis

**Affiliations:** 1 Health Sciences, Neapolis University of Pafos, Pafos, Cyprus; 2 Social Sciences, Hellenic Open University, Patras; 3 Psychology, Panteion University; 4 Psychology, Harokopeion University, Athens; 5 Psychology, University of Macedonia, Florina, Greece; 6 Health Sciences, Neapolis University Pafos, Pafos, Cyprus; 7 Psychology, National Kapodistrian University, Athens, Greece

## Abstract

**Introduction:**

According to international research, chronic diseases affect people’s life expectancy. There are many risk factors for Chronic Disease, both communicable and non-communicable. Chronic Disease can cause a variety of problems for the person suffering from it, such as physical, social and psychological distress. Therefore, patients’ coping strategies can affect their quality of life and the progression of the disease

**Objectives:**

This research aimed to investigate the relationship between Coping Strategies of patients with Chronic Diseases and their Psychological Distress experienced as a consequence of the disease.

**Methods:**

Survey participants were recruited via social media groups for chronic disease. So,106 people suffering from diseases, such as diabetes, arthritis, asthma, Multiple Sclerosis and other disabilities were involved in the study and were asked to respond to an internet-based questionnaire consisted of demographic questions and two scales: (1)Toulouse’s Scale for Coping, (2) Kessler Psychological Distress Scale (K6).

**Results:**

From the results derived by correlating specific parameters and factors such as gender, occupational status, marital status, educational level, place of residence and age, it was found that Chronic Disease’s management is related to psychological distress of patients. Namely, withdrawal and denial were associated with negative mental health state. Therefore, participants’ psychological distress and the strategies they chose to cope with their chronic illness were determined by a reciprocal relationship.

**Table 1:** correlations of coping strategies with psychological distress

*Correlations*					
	NERVOUS	DESPAIRED	RESTLESS OR HYPERACTIVE	NOTHING CAN MAKE YOU HAPPY	EVERYTHING NEEDED MORE EFFORT
FOCUS	.227^*^	.234^*^	0.155	.250^**^	.240^*^
SOCIAL SUPPORT	0.036	0.052	0.093	0.132	0.080
WITHDRAWAL	.536^**^	.466^**^	.418^**^	.551^**^	.457^**^
CHANGE	0.023	-0.177	-0.114	-0.009	0.035
CONTROL	-0.082	-0.156	-0.056	-0.083	0.082
DENIAL	0.167	0.173	0.183	.276^**^	.316^**^

**. Correlation is significant at the 0.01 level (2-tailed).

*. Correlation is significant at the 0.05 level (2-tailed).

**Conclusions:**

In light of the results, psychoeducational interventions aimed at alleviating psychological distress in patients with chronic diseases and improving their coping strategies are crucial.

**Disclosure of Interest:**

None Declared

